# Assessment of the nail penetration of antifungal agents, with different physico-chemical properties

**DOI:** 10.1371/journal.pone.0229414

**Published:** 2020-02-27

**Authors:** H. Davies-Strickleton, Julie Cook, Sally Hannam, Rhys Bennett, Alan Gibbs, David Edwards, Christine Ridden, John Ridden, David Cook

**Affiliations:** 1 Blueberry Therapeutics Limited, Alderley Park, Alderley Edge, Cheshire, United Kingdom; 2 Alderley Analytical Limited, Alderley Park, Alderley Edge, Cheshire, United Kingdom; Monash University Malaysia, MALAYSIA

## Abstract

Onychomycosis, or fungal nail infection, is a common fungal infection largely caused by dermatophyte fungi, such as *Trichophyton rubrum* or *Trichophyton mentagrophytes*, which affects a significant number of people. Treatment is either through oral antifungal medicines, which are efficacious but have significant safety concerns, or with topical antifungal treatments that require long treatment regimens and have only limited efficacy. Thus, an efficacious topical therapy remains an unmet medical need. Among the barriers to topical delivery through the nail are the physico-chemical properties of the antifungal drugs. Here, we explore the ability of a range of antifungal compounds with different hydrophilicities to penetrate the nail. Human nail discs were clamped within static diffusion (Franz) cells and dosed with equimolar concentrations of antifungal drugs. Using LC-MS/MS we quantified the amount of drug that passed through the nail disc and that which remained associated with the nail. Our data identified increased drug flux through the nail for the more hydrophilic compounds (caffeine as a hydrophilic control and fluconazole, with LogP -0.07 and 0.5, respectively), while less hydrophilic efinaconazole, amorolfine and terbinafine (LogP 2.7, 5.6 and 5.9 respectively) had much lower flux through the nail. On the other hand, hydrophilicity alone did not account for the amount of drug associated with/bound to the nail itself. While there are other factors that are likely to combine to dictate nail penetration, this work supports earlier studies that implicate compound hydrophilicity as a critical factor for nail penetration.

## Introduction

Onychomycosis (OM; fungal nail infection) is a common and contagious fungal infection of the nail plate and nail bed, leading to the gradual destruction of the nail plate [1]. The vast majority of cases of OM are caused by dermatophyte fungi. In 80–98% of affected individuals, *Trichophyton rubrum* or *Trichophyton mentagrophytes* are identified as the causative pathogen [2]. OM is considered the most prevalent of the nail ailments, accounting for about 50% of all diseased nails and about 30% of cutaneous fungal infections [1]. The prevalence of OM is reported to be 23% across Europe, 13.8% in North America and approximately 10% in Japan [3], with the prevalence increasing in Western countries, presumably due to lifestyle changes and ageing of the population [1].

The ‘gold standard’ treatment for OM is oral dosing with the antifungal terbinafine, but this treatment comes with a number of safety and tolerability issues, including rare cases of liver failure [4]. In contrast, topical antifungal treatments, such as, efinaconazole (10% nail solution, US), tavaborole (5% nail solution, US), ciclopirox olamine (8%), amorolfine, tioconazole, bifonazole/urea require long treatment times (>12 months) and only have modest efficacy rates. Topical therapies can be enhanced with chemical, mechanical or physical methods, however, these can cause other unwanted side-effects, such as tissue damage and pain [5]. Thus, there remains the need for a topical treatment with the efficacy of terbinafine but without the safety concerns of oral treatment or harsh topical enhancement strategies.

The difficulty in eradicating fungal nail infections by topical treatment is a consequence of factors intrinsic to the nail: the hard, protective nail plate, sequestration of pathogens between the nail bed and plate, and slow growth of the nail [6]. Furthermore, the unique barrier properties of the nail plate hamper the passage of antifungal drugs to deliver tissue concentrations required to eradicate the deeply seated causative fungi in the nail bed [7].

The physico-chemical properties of antifungal drugs also dictate their ability to penetrate the nail. Molecular weight, hydrophilicity, ionisation status and keratin binding capacity are all considered factors that affect the ability of topically applied drugs to penetrate the nail [7–9]. Of these properties, compound hydrophilicity is believed to be required for drugs to access the ‘hydrophilic pathway’ in order to penetrate and permeate through the nail [10–12]. This is supported by the observation that compounds with lower hydrophilicity show lower drug permeation and flux into and through the nail [3, 13].

Thus far, however, comparisons of the ability of drugs with different physico-chemical properties to penetrate the nail have been made using high concentration, saturated drug solutions/suspensions [13], or concentrations by weight [3]. In contrast, comparisons based on fixed molar concentration have not, to our knowledge, been reported. This differentiation would help to separate the influence of drug hydrophilicity versus solubility on drug permeation and flux, which may also provide a focus to facilitate the design of new drugs for the topical treatment of fungal nail infections.

In this short communication, we present our work, expanding on previous research, to explore the relationship between the hydrophilicity of antifungals and their nail penetration. We utilise static diffusion cells (Franz cells) with healthy human nail samples to measure the ability of a range of antifungal drugs (fluconazole, efinaconazole, amorolfine, terbinafine) with different degrees of lipophilicity (LogP) to penetrate the nail. We also used caffeine as a positive control for drug flux and permeation experiments [13]. Drugs were formulated in the same vehicle and at equivalent molar concentrations to enable direct comparisons of nail penetration without drug solubility in the applied solution being a factor. Drug concentrations associated with the nail itself and passing through the nail (drug flux) were determined by quantitative LC-MS/MS. These analyses confirm the relationship between the LogP of compounds and their ability to penetrate through the human nail and supports the hypothesis that water solubility and access to the ‘hydrophilic pathway’ are important factors for effective nail penetration of topically applied antifungal drugs.

## Materials and methods

### Ethics statement

Healthy nail clippings were obtained from healthy volunteers who provided their written consent. Nail samples from living persons are not classified as ‘relevant material’ under the Human Tissue Act 2004 [14], and the NHS Research Ethics Committee (REC) approval tool by the Health Research Authority [15] deemed that this study did not require approval.

### Materials

Caffeine, fluconazole, efinaconazole, amorolfine hydrochloride, terbinafine hydrochloride, fenpropimorph and flutriafol pestanal were obtained from Sigma Aldrich Ltd, UK. Caffeine-d9 and terbinafine-d7 were purchased from Toronto Research Chemicals, Canada. Acetonitrile and formic acid were purchased from Fisher Scientific, Loughborough, UK, and ultra-pure water from VWR, UK.

### Formulations for diffusion cells

Stock solutions of 20 mM were prepared in 100% (v/v) ethanol, except for caffeine, which was prepared at 20 mM in ultrapure water (UPW). Stock solutions were then diluted to yield 2 mM solutions with a final vehicle concentration of 20% (v/v) ethanol.

### Static diffusion (Franz) cell experiment set-up

**Fig 1:**
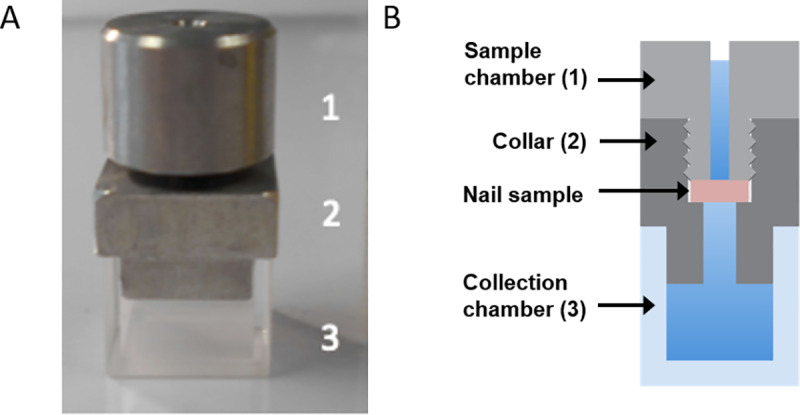
Static diffusion (Franz) cell. Photo (a) and schematic diagram (b) of a Franz cell, showing the sample chamber (1) and collar (2), made from stainless steel, and lower collection chamber (3) made of quartz. The nail sample was placed on a lip in the collar such that the upper surface of the nail was orientated upwards, the sample chamber was then screwed onto the collar, clamping the nail in place. Compounds were applied to the well created by the sample chamber and the top of the nail plate. The collection chamber was filled with UPW.

Our *ex vivo* static diffusion (Franz) cell experiment was adapted from established methods [3, 13, 16]. Nail clippings were soaked in UPW for 2 hours at 37 ^o^C prior to use and 3 mm discs cut from the nails using a biopsy punch tool. Nail discs were weighed and placed into the collar of a Franz cell (Fig 1; kindly donated by S. Murdan, University College London, UK) ensuring that they were in the appropriate orientation with the upper nail surface exposed to the sample chamber. The sample chamber was screwed down onto the nail until firmly fixed, ensuring that the nail disc covered the hole. The lower collection chamber was filled with 600 μL UPW and the well on the underside of the sample chamber-nail-collar assembly was filled with UPW to prevent bubbles forming beneath the nail. The sample chamber-nail-collar assembly was carefully placed into the collection chamber ensuring not to introduce any air bubbles. Excess liquid from the collection chamber was expelled at this point leaving a final volume of liquid in the lower chamber of 500 μL. A small amount of petroleum jelly was used to seal the collection chamber to the collar, while Parafilm (Fisher Scientific, Loughborough, UK) was used to wrap the join between the upper and lower chambers to prevent liquid evaporation.

The 2 mM caffeine or antifungal solutions, in a volume of 40 μL, were added into the sample chamber, ensuring not to introduce any air bubbles at the nail/liquid interface. Franz cells were incubated at 32 ^o^C in a humidified incubator for 7 days. Each compound was applied topically to 4 nail discs (caffeine) or 5 nail discs (antifungals).

### Collection of Franz cell samples to assess drug that penetrates the nail

Following incubation, the sample chamber-nail-collar assembly was carefully removed from the collection chamber. Any remaining topical solution was removed from the sample chamber and discarded. The sample chamber-nail-collar assembly was inverted, and the underside of the nail gently washed with 5 x 20 μL 50% (v/v) acetonitrile to remove any drug associated with the underside of the nail. The combined washes (underside of nail wash) were retained for analysis. The receptor fluid (liquid in the collection chamber) was collected for subsequent analysis. The collection chamber was washed with 100 μL 50% (v/v) acetonitrile (collection chamber wash). In this way, all of the compound that had passed through the nail could be identified by analysis of the following samples: underside of nail wash, collection chamber wash and receptor fluid. Receptor fluids and washes were stored at -20 ^o^C for several weeks prior to LC-MS/MS analysis for drug quantification (for LC-MS/MS methods see S1 File).

### Drug flux calculations

The amount of drug that passed through the nail was determined here by quantification of the receptor fluid, underside of nail wash and collection chamber wash from Franz cells. It was calculated that the surface area of nail in contact with the drug solution was 0.018 cm^2^. This was based on a 1.5 mm diameter circle of the 3 mm nail disk being in contact with the drug solution, whilst the remainder of the nail formed the seal with the upper chamber of the Franz cell.

### Collection of Franz cells samples to assess drug associated with the nail

Following the collection of samples from the collection chamber, the well created by the top of the nail and the sample chamber was washed with 5 x 100 μL UPW, with each wash being discarded, in order to remove any residual compound remaining in the sample chamber. The sample chamber and collar were disassembled, and the nail sample removed. The nail was washed by immersion in a large volume of UPW and dried using a clean tissue. The nails were then dissolved in 200 μL of 5 M NaOH at 37 ^o^C for 1 hour, and 200 μL methanol added prior to subsequent analysis [16]. Nail lysates were stored at -20 ^o^C for several weeks prior to LC-MS/MS analysis for drug quantification (for LC-MS/MS methods see S1 File).

## Results

### Nail penetration studied in Franz cells

A range of antifungals with different physico-chemical properties were assessed for their ability to penetrate the nail. Efinaconazole and fluconazole (azole antifungals), amorolfine (a morpholine) and terbinafine (an allylamine) were chosen to cover a range of hydrophobicity (lipophilicity), determined by their LogP values (Table 1). Luliconazole and ketoconazole antifungals were also considered for analysis but could not be studied due to poor solubility under the chosen conditions, while ciclopirox was not used due to known analytical challenges caused by chelating effects of ciclopirox with trace metal ions in chromatographic columns [17]. Caffeine was included in the assessment as a compound with high hydrophilicity that is known to have high drug flux through human nail ([13]; LogP -0.07, Table 1).

**Table 1:** Hydrophobicity (lipophilicity, LogP) and Molecular Weight (M_*w*_) of test compounds.

Compound	LogP	M_*w*_
Caffeine	-0.07	194
Fluconazole	0.5	306
Efinaconazole	2.7	348
Amorolfine	5.6	317
Terbinafine	5.9	291

LogP values were obtained from National Center for Biotechnology Information.

Healthy nail has previously been shown to demonstrate similar penetration to antifungal drugs as nails from onychomycosis patients [13], and so was used here for the assessment of *ex vivo* nail penetration. Caffeine and antifungal drugs were prepared to a fixed molar concentration (2 mM) in the same vehicle (20% (v/v) ethanol) and applied to healthy human nail discs clamped within Franz cells for 7 days at 32 ^o^C in a humidified incubator (Fig 1). After this time, the nail disc was washed and lysed to quantify the amount of compound associated with the nail, and the total amount of drug passing through the nail was quantified in receptor fluids and washes in order to calculate the drug flux.

### Association of drugs with nail

**Fig 2:**
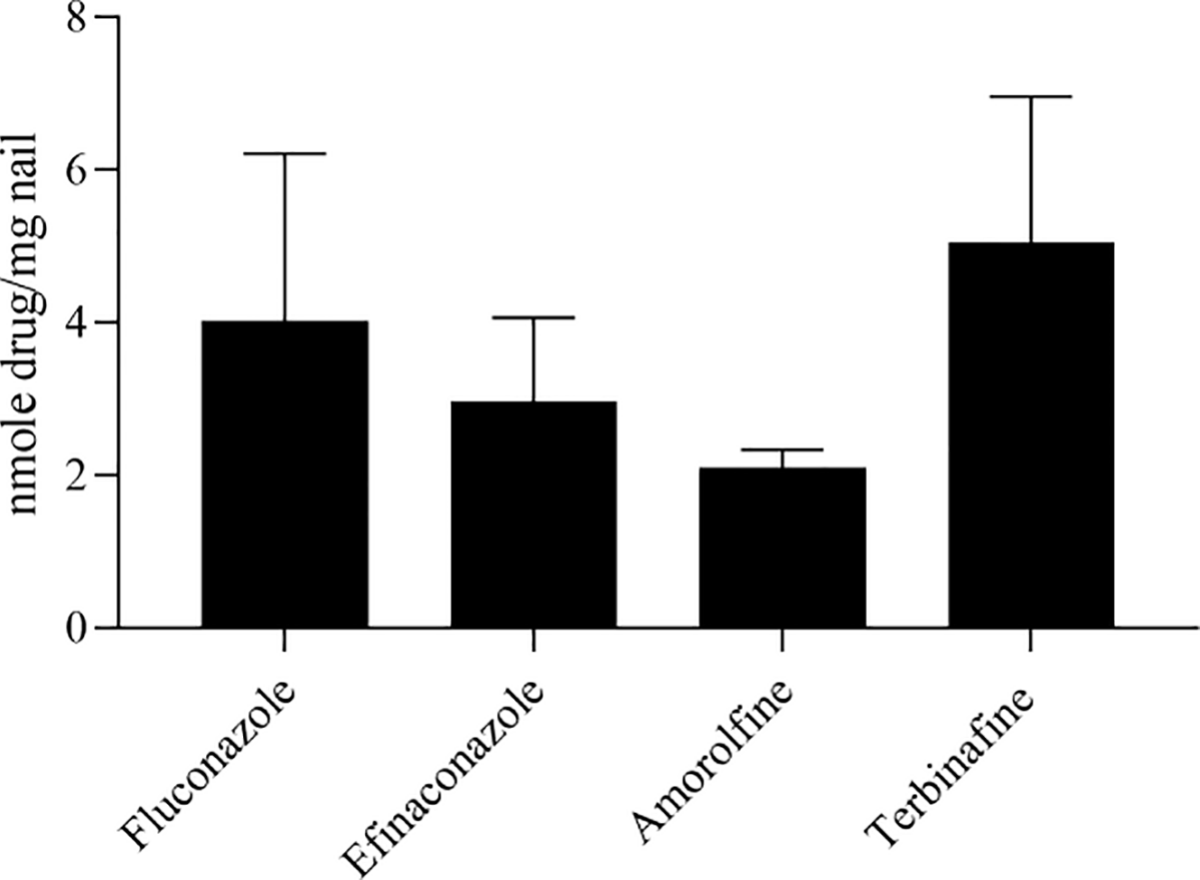
Quantification of compounds associated with nail samples. Nail lysates were prepared from nail discs and analysed by LC-MS/MS. Caffeine could not be identified due to lack of stability in 5 M NaOH used to lyse the nail (Table C in S1 File). Data were normalised to the weight of the individual nail samples. Error bars represent standard error of the mean of data from 4–5 different nails for each compound.

All nail lysates of nails from Franz cells treated with antifungal compounds contained drug concentrations above the Lower Limit of Quantification (LLoQ) in our LC-MS/MS analyses (1–10 ng/mL; Table B in S1 File). Quantification of nail lysates revealed that fluconazole (4.0 ± 2.2 nmole/mg), efinaconazole (3 ± 1.1 nmole/mg), amorolfine (2.1 ± 0.2 nmole/mg) and terbinafine (5.1 ± 1.9 nmole/mg) were found at relatively similar levels in the nail lysate samples (Fig 2). Statistical comparison by unpaired t-tests demonstrated that these levels were not statistically different from each other. Caffeine was not stable in the nail lysate matrix (Table C in S1 File) and so could not be quantified in these samples.

### Ability of drugs to penetrate the nail

Drug concentrations were quantified under the nail in receptor fluids and washes of the underside of nail and collection chamber. Concentrations in receptor fluids were at least 5–10 fold lower than the water solubility of compounds (Table D in S1 File), demonstrating that sink conditions were met, and that drug permeation was not limited by drug saturation under the nail. Due to the aqueous nature of the receptor fluid, organic solvent (50% (v/v)) was used to wash the underside of the nail and collection chamber to facilitate full recovery of drug under the nail.

**Fig 3:**
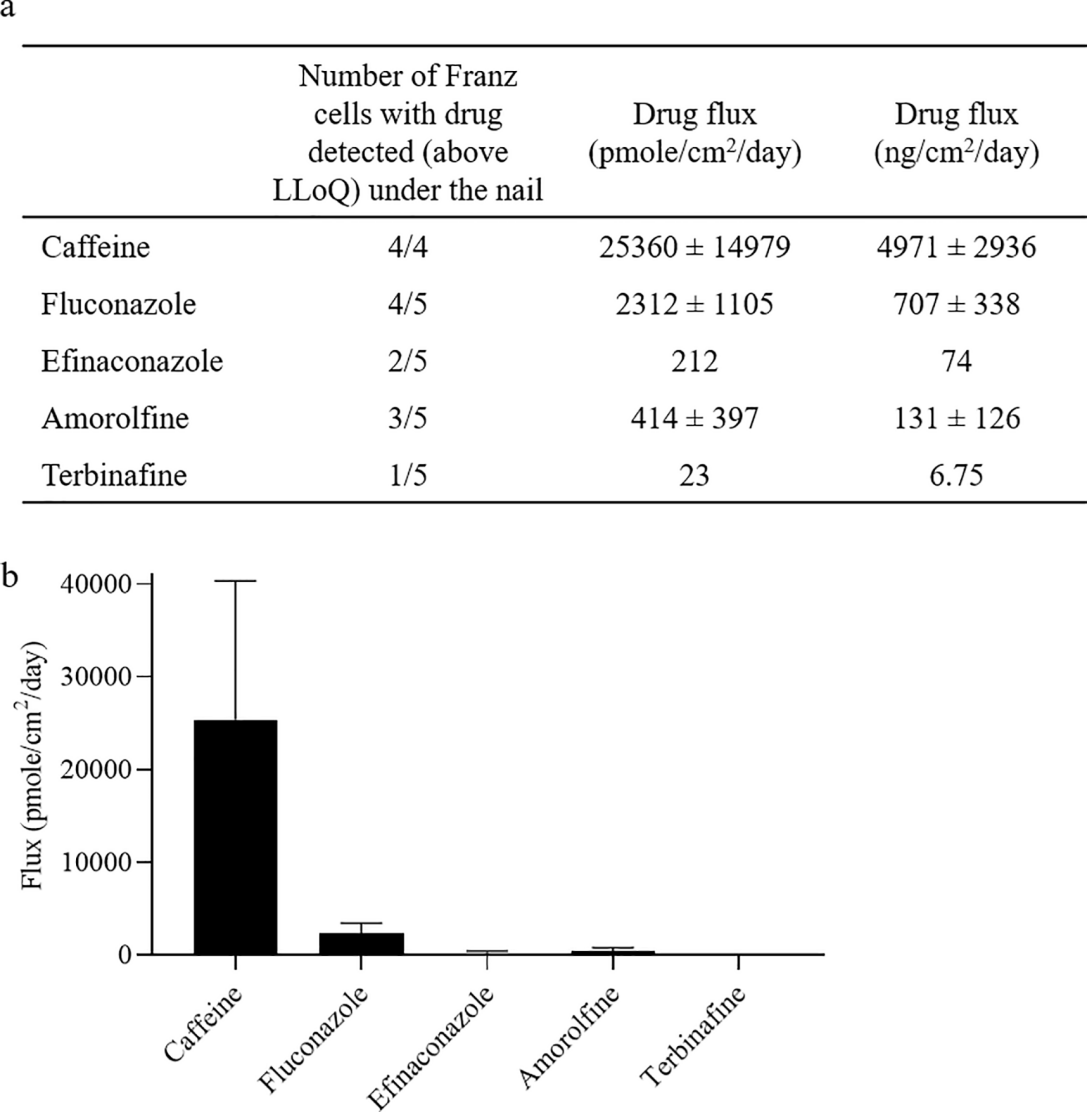
Total drug that passed through the nail. (a) The number of Franz cells in which drug was detected to have passed through the nail. (b) Drug flux through the nail. Error bars representing standard error of the mean are shown for compounds where at least 3 values were above the LLoQ.

The number of Franz cells in which drug was detected above LLoQ (1–2 ng/mL, Table B in S1 File) under the nail (in either the receptor fluid, the underside of nail wash or the collection chamber wash) was different for the various compounds: 4/4 for caffeine, 4/5 for fluconazole, 2/5 for efinaconazole, 3/5 for amorolfine and 1/5 for terbinafine (Fig 3A). For those Franz cells that were detected above LLoQ, drug flux was calculated, and was found to be highest for caffeine (25360 ± 14979 pmole/cm^2^/day), and lower for the antifungal drugs: fluconazole (2312 ± 1105 pmole/cm^2^/day), efinaconazole (212 pmole/cm^2^/day), amorolfine (414 ± 397 pmole/cm^2^/day), terbinafine (23 pmole/cm^2^/day; Fig 3B). Statistical t-testing was not performed to compare drug flux due to various datapoints being below the limit of quantification.

Comparison of drug flux with the physico-chemical properties of the compounds (Table 1) suggests that there is trend between drug flux through the nail and molecular hydrophilicity. Caffeine, the most hydrophilic molecule tested (LogP, -0.07), showed the largest drug flux (25360 ± 14979 pmole/cm^2^/day), and fluconazole (LogP, 0.5; the most hydrophilic antifungal tested) had the highest drug flux of the antifungal compounds (2312 ± 1105 pmole/cm^2^/day). Efinaconazole, amorolfine and terbinafine are less hydrophilic (LogP 2.7, 5.6 and 5.9, respectively) and were below the limit of quantification in more of the Franz cells, whilst those above LLoQ showed much lower drug flux (212 pmole/cm^2^/day, 414 ± 397 pmole/cm^2^/day and 23 pmole/cm^2^/day, respectively; Fig 3). Multi-linear regression analysis demonstrated that the correlation between drug flux and LogP was statistically significant (p = 0.027), while nail lysate concentration and LogP were not statistically significant (Figure C in S1 File).

## Discussion

In this short communication, we report the nail association and permeation of compounds with different physico-chemical properties through healthy human nail. Our data indicate a large difference in the ability of caffeine, fluconazole, efinaconzole, amorolfine and terbinafine to permeate through human nail. This difference correlated with molecular hydrophilicity, with greater hydrophilicity corresponding to greater nail permeation. In contrast, the amount of compound associated with the nail did not differ greatly between compounds nor correlate with molecular hydrophilicity.

Using LC-MS/MS we performed quantitation to examine nail lysate concentrations and drug flux through the human nail under conditions in which dose concentration and vehicle were consistent and well-defined. This approach of using equimolar drug concentrations in a fixed vehicle to examine parameters such as molecular hydrophilicity is to our knowledge novel and has enabled here the direct comparison of the ability of antifungals with different physico-chemical properties to penetrate the nail. This builds on the work of other research groups in which physico-chemical properties have been explored using saturated drug concentrations, or concentrations by weight [3, 13]. By removing these other variables from our experimental design, we have demonstrated with greater clarity the importance of molecular hydrophilicity in nail permeation.

Here, we showed that the compounds tested exhibited large differences in their ability to permeate the nail (Fig 3). All Franz cells treated with caffeine showed relatively high drug flux through the nail (25360 ± 14979 pmole/cm^2^/day). Fluconazole was also detected under the nail in the majority of Franz cells treated with this compound, with lower drug flux than caffeine (2312 ± 1105 pmole/cm^2^/day). In contrast, efinaconazole, amorolfine and terbinafine were detected under the nail in fewer Franz cells and drug flux was far lower (212, 414 ± 397 and 23 pmole/cm^2^/day, respectively), demonstrating that they did not pass as readily through the nail as fluconazole and caffeine. Importantly, since the drug concentrations detected under the nail were at least 5–10 x lower than the water solubility of compounds (Table D in S1 File), sink conditions were met, demonstrating that drug flux was not limited by drug saturation under the nail.

The drug flux data and trends amongst compounds reported here are comparable to those observed elsewhere. In previous work, McAuley et al. reported greater flux through healthy and onychomycotic nails for caffeine compared to amorolfine and terbinafine [13], and Matsuda et al. reported that efinaconazole had a greater ability to penetrate the nail than amorolfine and terbinafine [3]. These observations are in agreement with those made in this study. Quantitatively, however, values reported by Matsuda et al. showed differences to the data reported here. For instance, Matsuda et al. did not detect any amorolfine or terbinafine under the nail [3], while we identified very low amounts under some of the nails tested. Furthermore, Matsuda et al. measured a drug flux through the nail for efinaconazole of 3.17 ng/cm^2^/day after 7 days [3], which is lower than that measured here (74 ng/cm^2^/day). These differences may be due to inherent differences in nail permeability between nail samples. Indeed, it has been noted previously that drug flux values are highly variable, which likely reflects differences in the barrier properties of individual nail samples [3]. Secondly, here we are likely to have overestimated drug flux as a result of some measurements below the limit of quantification. Here, the LLoQ was 2 ng/mL for efinaconazole in receptor fluids and washes, equivalent to an LLoQ for drug flux of 1.6 ng/cm^2^/day, and data below this were not detected and so could not be taken into account when calculating mean drug flux. Thirdly, differences in experimental design, such as vehicle (20% (v/v) ethanol here, compared to propylene glycol: ethanol 1:4 (v/v) used by Matsuda et al. [3]) and drug concentration may impact the solubility and availability of the compounds, altering their ability to permeate the nail.

Our data also demonstrated that drug flux correlated with molecular hydrophilicity. For instance, the drug flux of caffeine was ~10-fold higher than fluconazole, ~100-fold greater than efinaconazole, ~60-fold greater than amorolfine and ~1000-fold greater than terbinafine (LogP -0.07, 0.5, 2.7, 5.6 and 5.9, respectively). Furthermore, these data suggest that even small changes in molecular hydrophilicity can have large impact on drug flux, exemplified by the 10-fold reduction in drug flux over a small LogP range (caffeine to fluconazole LogP -0.07–0.5). Multi-linear regression analysis confirmed that the correlation between drug flux and hydrophilicity was statistically significant (Figure C in S1 File). Thus, our findings implicate molecular hydrophilicity as an important determinant in the ability of compounds to permeate the nail.

This finding supports and builds upon previous studies that have suggested a role of hydrophilicity in nail permeation [3, 13]. Although strongly implicating a role of molecular hydrophilicity in the ability of drugs to permeate the nail, previous work utilised saturated drug solutions/suspensions [13], or concentrations by weight [3], making it difficult to ascertain the extent to which hydrophilicity, as opposed to drug solubility, influenced drug permeation and flux. The utilisation of consistent and well-controlled dose concentration and vehicle here has enabled the importance of hydrophilicity to be revealed. Furthermore, by incorporating an additional antifungal, fluconazole, and demonstrating its greater ability to penetrate the nail than less-hydrophilic efinaconazole, amorolfine and terbinafine, but not to the same level as more-hydrophilic caffeine, we have added greater support to the role of hydrophilicity for nail penetration.

In this study, we focussed on compounds that differed in their hydrophilicity, but it is important to note that other molecular properties influence their ability to permeate the nail. For instance, it has been observed that molecules of around 200 Da have greater nail penetration than those of around 300 Da [18]. Indeed, this may also contribute to the far greater drug flux of caffeine (196 Da) compared to the antifungals (291–348 Da) seen here. Examination of compounds with a broader range of MW than we researched here would be needed to explore this further.

In contrast to drug flux through the nail, the level of drug associated with the nail itself did not show great differences between compounds. All the antifungal compounds showed similar levels of drug within the nail lysates, which represented both drug within the nail and drug bound to the nail’s upper surface (Fig 1). Multi-linear regression analysis confirmed that there was no correlation of statistical significance between LogP and the levels of drugs within nail lysates (Figure C in S1 File). These data suggest that drug association with the nail *per se* is not a good predictor of nail permeation, and that molecular hydrophilicity alone does not account for the levels present bound to and within the nail. Indeed, other factors are likely to influence both the permeation and association of molecules with the nail. In addition to compound MW, keratin-binding capacity has been noted elsewhere as an important barrier to the movement of a compound through the nail [3, 9], as well as compound ionisation status, which influences its LogP [8].

While molecular hydrophilicity is highlighted here as a predictor of drug flux, it is noteworthy that drug flux and transit across the nail following topical application are not the only important factors for treating nail diseases, such as OM. For instance, despite its superior ability to penetrate the nail, fluconazole is not as potent against OM-causing dermatophytes as terbinafine [19], which showed the lowest nail permeation here. Therefore, both nail permeation and drug efficacy will be important features of a successful topical agent to target nail diseases.

## Conclusion

The data reported here strongly support a relationship between nail penetration and hydrophilicity for anti-fungal drugs. The observations here, and supported elsewhere [13], suggest that water solubility and access to the ‘hydrophilic pathway’ appears to be a major determinant of drug flux through the nail. However, it is likely that various properties (hydrophilicity, molecular weight, keratin binding capacity and ionisation status) act in concert to determine the ability of a drug to permeate the nail. Ultimately, a balance between drug potency and nail permeation will be required for the development of a successful topical therapy to OM.

## Supporting information

S1 File Table A. UPLC gradient for compound elution.
Table B. Transitions and lower limit of quantification (LLoQ) for compounds by LC-MS/MS analysis (ng/mL).
Figure A. Example chromatograms from LC-MS/MS analysis.
Figure B. Example calibration curve from LC-MS/MS analysis.
Table C. Stability of compounds after sample preparation.
Table D. Drug concentrations in receptor fluids.
Figure C. Multi-linear regression analysis of drug flux and nail lysate concentration versus LogP.
(DOCX)
