# Supplementary material for: Assessment of the nail penetration of antifungal agents, with different physico-chemical properties
Source: PLoS One. 2020 Feb 27;15(2):e0229414. doi: 10.1371/journal.pone.0229414 (PMC7046211; doi:10.1371/journal.pone.0229414)
Supplement: S1 File — Table A. UPLC gradient for compound elution. Table B. Transitions and lower limit of quantification (LLoQ) for compounds by LC-MS/MS analysis (ng/mL). Figure A. Example chromatograms from LC-MS/MS analysis. Figure B. Example calibration curve from LC-MS/MS analysis. Table C. Stability of compounds after sample preparation. Table D. Drug concentrations in receptor fluids. Figure C. Multi-linear regression analysis of drug flux and nail lysate concentration versus LogP. (DOCX) [file pone.0229414.s001.docx]

Supplementary information

# Assessment of the nail penetration of antifungal agents, with different physico-chemical properties

Davies-Strickleton Heather^1^, Cook Julie^1^, Hannam Sally^2^, Bennett Rhys^2^, Gibbs Alan^2^, Edwards David^1^, Ridden Christine^1^, Ridden John^1^, Cook David^1*^

^1^Blueberry Therapeutics Limited, Alderley Park, Alderley Edge, Cheshire, UK

^2^ Alderley Analytica Limitedl, Alderley Park, Alderley Edge, Cheshire, UK

* Corresponding author email: David.cook@blueberrytherapeutics.com

Supplementary methods

Preparation of Franz cell samples for quantitation

Receptor fluids were diluted in an equivalent volume of acetonitrile to yield samples in 50 % (v/v) acetonitrile. Diluted receptor fluids and wash samples were quantified using calibration standards (1, 2, 5, 10, 50, 100, 300, 600, 1000 and 5000 ng/mL) for caffeine, fluconazole, efinaconazole, amorolfine hydrochloride and terbinafine hydrochloride prepared in 50 % (v/v) acetonitrile. Quality control samples were prepared to 20, 200 and 800 ng/mL. Samples and standards were prepared for measurement by diluting 1 in 20 with 50 % (v/v) acetonitrile and addition of a final concentration of 100 ng/mL internal standard. The following internal standards were used: caffeine d-9 (for caffeine); fenpropimorph (for amorolfine); flutriafol pestanal (for efinaconazole and fluconazole); and terbinafine-d7 (for terbinafine).

Nail lysate samples were quantified using calibration standards (1, 2, 5, 10, 50, 100, 300, 600, 1000 and 5000 ng/mL) prepared in 1:1 methanol:blank nail lysate in 5 M NaOH. This method accounted for the reduced stability of antifungals that was observed in 5 M NaOH compared to 50 % acetonitrile (Table C). Samples and standards were prepared for measurement by diluting 1 in 30 with 50 % (v/v) acetonitrile and addition of a final concentration of 100 ng/mL internal standard.

Quantitation of Franz cell samples by LC-MS/MS

Ultra-performance liquid chromatography (UPLC) was performed on a Waters (Milford, MA, USA) iClass Acquity system, using a Kinetex XB C18 (Phenomenex, Torrance, CA, USA) analytical column (50 x 2.1 mm, 2.6 µm). Mobile phases were 0.1 % aqueous formic acid (A) and 0.1 % formic acid in acetonitrile (B). The column temperature was set at 45 ^o^C and compounds were eluted at a flow rate of 0.6 mL/min using the gradient shown in Table A.

## Mass spectrometry was performed on a Waters TQ-D triple quad mass spectrometer with electrospray ionization (ESI). It was operated in positive ion mode with a source temperature of 150 °C, desolvation temperature 500 °C, desolvation gas flow 800 L/h, capillary voltage 3 kV, cone voltage 35 V, cone gas flow 150 L/h and collision energy 20 V. Data were processed using Waters UNIFI software, version 1.9. Transitions were measured as shown in Table B, together with limit of quantification for each compound. Example chromatograms and calibration curves are shown in Figures A-B.

Testing compound stability in matrices

Compounds were prepared to 200 µM in either 50 % (v/v) acetonitrile or 5 M NaOH for comparison of stability of wash samples and diluted receptor fluids, and nail lysates, respectively. Those in 5 M NaOH were also were heated at 37 ^o^C for 1 hour to mimic sample preparation of the nail lysate. Samples were stored at -20 ^o^C for several weeks, diluted to 2 µM in 50 % (v/v) acetonitrile and analysed by LC-MS on a Waters Acquity coupled with Mass detector QDA. Results indicated a reduction of stability for the antifungal compounds that had been exposed to 5 M NaOH compared to 50 % acetonitrile (Table C), therefore, for the quantification of compounds in the nail lysates, calibration standards were prepared in 5 M NaOH as well. Caffeine was not stable in 5 M NaOH and so could not be quantified in nail lysate samples. For quantification of receptor fluids and washes, 50 % (v/v) acetonitrile was chosen to match the matrix of these samples. Data are shown in Table C.

Multi-linear regression analysis

Drug flux data were compared to LogP by multi-linear regression analysis using JMP software version 15.0 from SAS. Where samples were below the limit of quantification for drug flux, the lowest detectable value in the dataset was used. This approach, while leading to slight overestimation of drug flux, enables statistical comparison to be performed. Confidence intervals were set to 95 %.

Supplementary results

**Table A: UPLC gradient for compound elution**.

| Time (min) | Composition A (%) | Composition B (%) |
| --- | --- | --- |
| 0.0 | 95 | 5 |
| 0.2 | 95 | 5 |
| 1.5 | 5 | 95 |
| 1.7 | 5 | 95 |
| 1.8  2.5 | 95  95 | 5  5 |

**Table B. Transitions and lower limit of quantification (LLoQ) for compounds by LC-MS/MS analysis.**

|  |  |  | LLoQ (ng/mL) | |
| --- | --- | --- | --- | --- |
| Compound | Precursor Ion | Product Ion | Receptor fluids and washes | Nail lysates |
| Caffeine | 195.1 | 137.8 | 2 | *ND |
| Fluconazole | 307.2 | 220.2 | 1 | 10 |
| Efinaconazole | 349.2 | 280.1 | 2 | 5 |
| Amorolfine | 318.3 | 161.1 | 1 | 2 |
| Terbinafine | 292.2 | 141.0 | 1 | 1 |
| Caffeine-d9 | 204.2 | 144.1 | n/a | n/a |
| Terbinafine-d7 | 299.2 | 148.0 | n/a | n/a |
| Flutriafol | 302.23 | 123.0 | n/a | n/a |
| Fenpropimorph | 304.48 | 147.0 | n/a | n/a |

*ND (not determined; caffeine was not stable in 5 M NaOH and so could not be quantified.)


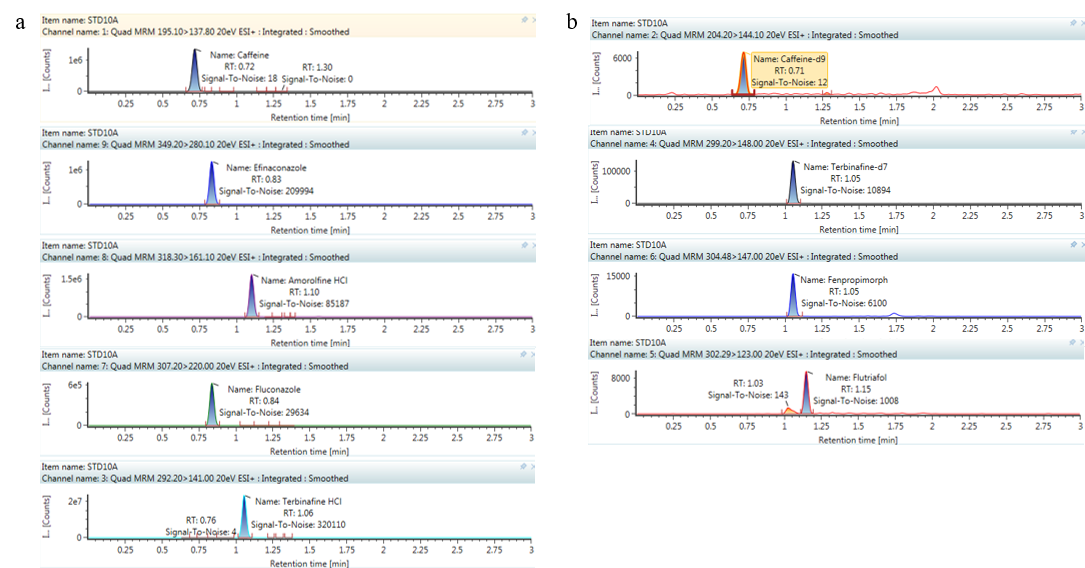


**Figure A. Example chromatograms from LC-MS/MS analysis.** Chromatograms of transititions for the analytes (a; caffeine, efinaconazole, amorolfine, fluconazole, terbinafine) and internal standards (b; caffeine-d9, terbinafine-d7, fenpropimorph, flutriafol).


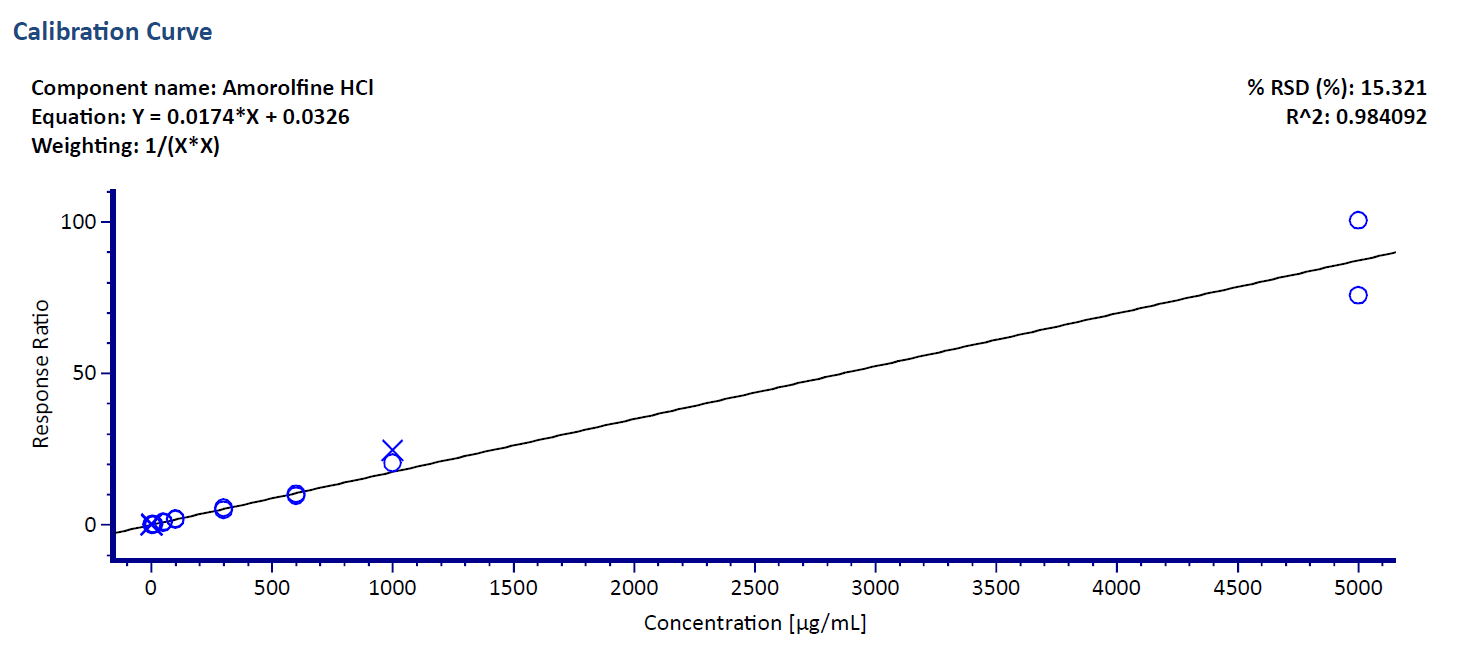


**Figure B. Example calibration curve from LC-MS/MS analysis.** Data are shown for duplicate standard concentrations of amorolfine in prepared in blank nail lysate matrix.

**Table C. Stability of compounds after sample preparation.** Compounds were prepared to 200 µM in either 50 % (v/v) acetonitrile or 5 M NaOH, stored at -20 ^o^C and later analysed by LC-MS/MS. Data were normalized to those obtained for 50% (v/v) acetonitrile. Standard error of the mean on 3 replicate stability experiments is shown.

| Compound | % recovery in 5 M NaOH |
| --- | --- |
| Caffeine | 0.02 ± 0.02 |
| Fluconazole | 55.3 ± 3.1 |
| Efinaconazole | 47.6 ± 29.5 |
| Amorolfine | 29.6 ± 2.2 |
| Terbinafine | 38.6 ± 4 |

**Table D. Drug concentrations in receptor fluids**

| Compound | Number of receptor fluids with drug detected above LLoQ | Concentration in receptor fluid (pM) | Water solubility (pM) |
| --- | --- | --- | --- |
| Caffeine | 4/4 | 6224 ± 3678 | 56 x 10^9^ |
| Fluconazole | 4/5 | 571 ± 273 | 4.5 x 10^9^ |
| Efinaconazole | 1/5 | 101 | 1.75 x 10^9^ |
| Amorolfine | 1/5 | 299 | 2.5 x 10^6^ |
| Terbinafine | 1/5 | 6 | 2.5 x 10^6^ |

Caffeine and fluconazole concentrations show mean values and standard error of the mean.


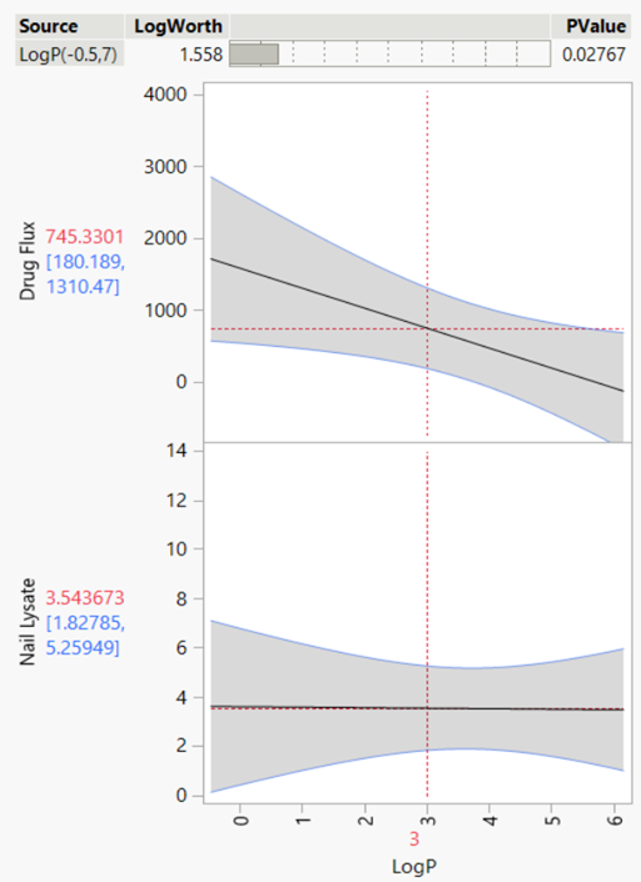


**Figure C. Multi-linear regression analysis of drug flux and nail lysate concentration versus LogP.** Confidence intervals were set at 95 %. The correlation between drug flux and LogP had a p value of 0.027.
